# Primary Progressive Multiple Sclerosis—A Key to Understanding and Managing Disease Progression

**DOI:** 10.3390/ijms25168751

**Published:** 2024-08-11

**Authors:** Izabela Sempik, Edyta Dziadkowiak, Helena Moreira, Anna Zimny, Anna Pokryszko-Dragan

**Affiliations:** 1 Department of Neurology, Regional Hospital in Legnica, Iwaszkiewicza 5, 59-220 Legnica, Poland; sempikiza2008@gmail.com; 2 Clinical Department of Neurology, University Centre of Neurology and Neurosurgery, Faculty of Medicine, Wroclaw Medical University, Borowska 213, 50-556 Wroclaw, Poland; anna.pokryszko-dragan@umw.edu.pl; 3 Department of Basic Medical Sciences, Wroclaw Medical University, Borowska 211, 50-556 Wroclaw, Poland; helena.moreira@umw.edu.pl; 4 Department of General and Interventional Radiology and Neuroradiology, Wroclaw Medical University, Borowska 213, 50-556 Wroclaw, Poland; anna.zimny@umw.edu.pl

**Keywords:** primary progressive multiple sclerosis, immune dysregulation, neurodegeneration, remyelination failure, biomarkers of progression

## Abstract

Primary progressive multiple sclerosis (PPMS), the least frequent type of multiple sclerosis (MS), is characterized by a specific course and clinical symptoms, and it is associated with a poor prognosis. It requires extensive differential diagnosis and often a long-term follow-up before its correct recognition. Despite recent progress in research into and treatment for progressive MS, the diagnosis and management of this type of disease still poses a challenge. Considering the modern concept of progression “smoldering” throughout all the stages of disease, a thorough exploration of PPMS may provide a better insight into mechanisms of progression in MS, with potential clinical implications. The goal of this study was to review the current evidence from investigations of PPMS, including its background, clinical characteristics, potential biomarkers and therapeutic opportunities. Processes underlying CNS damage in PPMS are discussed, including chronic immune-mediated inflammation, neurodegeneration, and remyelination failure. A review of potential clinical, biochemical and radiological biomarkers is presented, which is useful in monitoring and predicting the progression of PPMS. Therapeutic options for PPMS are summarized, with approved therapies, ongoing clinical trials and future directions of investigations. The clinical implications of findings from PPMS research would be associated with reliable assessments of disease outcomes, improvements in individualized therapeutic approaches and, hopefully, novel therapeutic targets, relevant for the management of progression.

## 1. Introduction

Multiple sclerosis (MS) is a long-lasting, multifocal demyelinating disease of the central nervous system (CNS). MS-related lesions, developing in the course of the disease, are disseminated throughout the brain and spinal cord and result in a wide range of symptoms (i.a., motor deficit, sensory impairment, visual deficit, bladder dysfunction or cognitive decline), which contribute to accumulating disability and substantially affect psychosocial aspects of patients’ functioning and their life quality [1]. The complex etiology of MS comprises autoimmune/inflammatory and neurodegenerative components. Originally, exacerbating immune-mediated demyelination was assumed as the background for early stages of the disease, clinically manifested in relapsing–remitting course, followed by neurodegeneration underlying the later, progressive stage [1]. Due to current research concepts, acute events of focal CNS injury are superimposed on the long-term process of progressive damage to CNS, slowly expanding throughout disease stages (“smouldering MS”) [2]. The pathology of this progressive damage, apart from the neurodegenerative process, also involves chronic and diffuse inflammation and failure of the repair capacity [3]. The emerging neurological deficit and its dynamics result from an interplay of relapses and progression across the entire range of the MS course [4].

Recent progress in understanding MS patophysiology has allowed substantial improvements in diagnostic and therapeutic opportunities. Several disease-modifying therapies (DMTs) are available, which can effectively control MS activity. However, the prevention and management of progression still pose a challenge to clinicians and thus remain subjects of extensive research.

Primary progressive multiple sclerosis (PPMS) is the least frequent type of the disease, characterized by a specific course and clinical symptoms, and associated with a poor prognosis. It requires extensive differential diagnosis and often a long-term follow up before its correct recognition. Due to this specificity, PPMS has sometimes been suggested to be a separate clinical entity. On the other hand, pathologic findings in CNS tissues and corresponding lesions shown in magnetic resonance imaging (MRI) of the brain and spinal cord are similar in patients with PPMS and the secondary progressive type of disease (SPMS), evolving from the relapsing–remitting one. Considering the modern concept of “smoldering” progression, already present but occult in the early stage of disease, a thorough exploration of PPMS may provide a better insight into mechanisms of progression in MS, with potential clinical implications.

The goal of this study was to review the current evidence from the investigation of PPMS, in the more general context of disease progression. We aimed at the integration of recent findings about cellular and molecular mechanisms underlying the PPMS background with a wide panel of clinical aspects, including diagnostic issues, potential monitoring and prognostic biomarkers and therapeutic opportunities.

## 2. Method—Literature Search

See: Supplementary Materials.

## 3. Terms and Definitions

The standardized definitions for clinical courses of MS, widely used in clinical practice, included relapsing–remitting (RRMS), secondary progressive (SPMS) and primary progressive (PPMS) type of disease, with the relapsing–progressive (RPMS) type usually distinguished during transition between RRMS and SPMS. On the basis of further research findings and clinical observations, a new classification was proposed in 2013 [5]. According to this, the two main phenotypes of MS (relapsing and progressive) can be additionally modified by the temporary presence of activity or progression. Thus, the active progressive type of MS is characterized by occasional relapses and/or new CNS lesions shown in MRIs, superimposed on periods of stable condition or the gradual accumulation of disability.

Furthermore, two types of clinical deterioration have been distinguished throughout all stages of the disease: relapse-associated worsening (RAW)—an increase in neurological deficit emerging from recent exacerbation—and progression independent of relapses (PIRA), slowly developing in the long-term course [4,6]. Data from clinical trials indicate that PIRA is responsible for 70–90% of all disability accrual events within 2–10 years of follow up [7,8]. Due to the variable dynamics of the disease, clinical worsening (especially RAW) may be temporary (with symptoms at least partly resolved after treatment) or persistent. Confirmed/sustained disability worsening, often used as the outcome measure in clinical trials, is usually defined as the increase in disability level (assessed in dedicated scales), which persists without improvement within three or six months of follow-up.

## 4. Primary Progressive Multiple Sclerosis (PPMS)

### 4.1. Clinical Characteristics

PPMS is the least common type of disease, diagnosed in about 10–15% of MS patients. Unlike with RRMS, both men and women are equally affected, and the mean age at onset is usually older (between 37 and 43 years). The familial rate of PPMS occurrence tends to be lower than in the overall MS population [9].

The onset of symptoms is often difficult to discern because of their insidious and slow development over time. Thus, the identification of progression is often retrospective, and the diagnostic process may be challenging. The most common clinical manifestation at onset includes motor deficit and sensory impairment with a pattern suggestive of myelopathy, often accompanied with bowel and bladder dysfunction. Cerebellar and brainstem symptoms may occur at early or later stage of disease, while optic neuritis is quite uncommon. Early and gradual deterioration in cognitive performance is often observed in PPMS [9,10].

Less than 30% of PPMS patients experience a distinct relapse throughout the disease (active type of PPMS), so the clinical worsening almost exclusively results from progression. However, its rate seems more rapid than in RRMS, with a shorter median time to milestones in the Expanded Disability Status Scale (EDSS) [11], corresponding with severe disability (mobility limitation, using the wheelchair). The initial increase in disability and the number of neurological functional systems involved at early stage of PPMS predict further rate of progression and time of survival [10].

Patients with PPMS have fewer and smaller brain lesions revealed in T2/FLAIR MR sequences but tend to have more lesions in the spinal cord. The inflammatory component is less prominent in PPMS; thus, gadolinium contrast-enhanced (Gd+)-lesions (active plaques associated with profound leakage of the blood–brain barrier) also occur much less frequently. On the contrary, diffuse atrophy of the brain and spinal cord is extensive and develops faster than in RRMS. Advanced neuroimaging techniques allow more specific aspects of PPMS-related CNS damage to be revealed [9,10].

The presence of oligoclonal IgG bands (OCBs) in the cerebrospinal fluid (CSF) and their absence in serum, indicating intrathecal IgG synthesis (OCB type 2), is considered typical for MS, both of relapsing–remitting and progressive types. OCBs in the CSF are present in up to 90% of patients with PPMS, and they have been included in the current version of McDonald diagnostic criteria for this type of disease [12]. However, Villar et al. [13] showed that in the majority of patients with PPMS, additional oligoclonal immunoglobulin G bands (OCGBs) can be found also in the serum (type III), unlike in those with RRMS and SPMS. Similar findings were observed in late-onset MS. It was suggested that this phenomenon may be due to the greater likelihood of systemic infections and blood–CSF barrier dysfunction in older patients, therefore related rather to aging than disease-specific mechanism [13,14,15].

### 4.2. Diagnostic Criteria

According to the separate chapter of the 2017 revision to the McDonald diagnostic criteria for MS [16], the recognition of PPMS requires confirmation of continued clinical progression, independent of relapse activity, observed retrospectively or prospectively for at least one year, with concomitance of at least two of the following categories: ≥one lesion detected in MRI of the brain (in typical regions: periventricular, (juxta)cortical, infratentorial), ≥two lesions detected in MRI of the spinal cord and presence of OCB in CSF.

Exclusion of alternative diagnoses can be particularly demanding challenge in case of PPMS.

Differential diagnosis should include CNS diseases with progressive course and predominant involvement of the spinal cord, cerebellum and brainstem. Among hereditary disorders with late onset, familial spastic paraparesis, X-linked adrenoleukodystophy, Wilson’s disease and hereditary ataxias should be taken into account. A similar clinical manifestation may occur in infectious (syphilis, brucellosis, HIV or HTLV-1-associated CNS involvement) or other systemic inflammatory disorders (neurosarcoidosis, lupus erythematosus, or Behcet disease), as well as in neurodegenerative diseases (multiple system atrophy). Finally, metabolic disturbances (e.g., vit. B12 deficiency) should be excluded [9,17,18].

## 5. PPMS—Background for Progression

**Figure 1:**
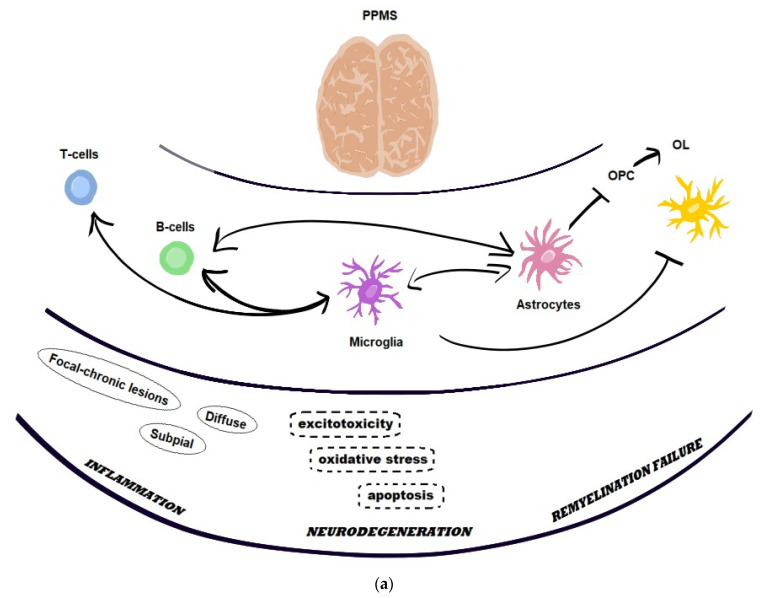

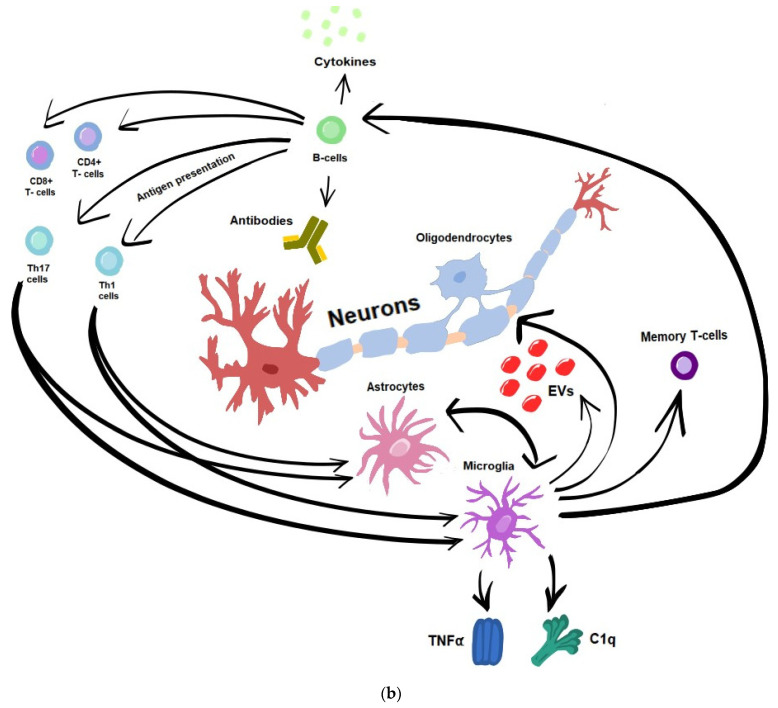
Background for disease progression in PPMS: (**a**) basic components, (**b**) key players involved in neuroinflammation and neurodegeneration.

Pathomechanisms involved in the background for CNS damage in PPMS comprise chronic inflammation, neurodegeneration with axonal loss and the failure of repair/compensation processes. Immunocompetent cells (T- and B-cells, NK) and their interactions with the cells residing in the CNS (such as astrocytes and microglia) contribute to inflammatory components, while the dysfunction of glial cells is also associated with neurodegeneration (including oxidative stress, excitotoxicity and apoptosis), and impaired proliferation and differentiation of oligodendrocytes results in remyelination failure [19,20,21] (Figure 1).

In pathological CNS findings from PPMS patients, chronic demyelinating lesions are predominant and mostly inactive, although some may display radial expansion and signs of activity, including the peripheral infiltration of microglia and macrophages, with demyelination at their edges. The specific types of lesions are characterized as smoldering or slowly expanding ones (with a thin rim of activated microglia ingesting iron and few myelin-containing macrophages) and shadow plaques, indicating partial remyelination. These lesions can be detected in white and gray matter, with cortical location regarded as most typical and correlating with clinical manifestation in PPMS. With the duration of the disease, the number of inactive lesions tends to increase, but their size decreases [22].

### 5.1. Immune-Mediated Inflammation and Neurodegeneration

Neuroinflammation in PPMS seems to be less dependent on systemic immune activity and compartmentalized within the CNS. Apart from focal chronic injury within demyelinative lesions, there are inflammatory infiltrates mainly located in the leptomeninges and diffuse alterations in apparently normal white and grey matter [22]. The key players of these processes include T- and B-cells, while CNS-resident cells provide links between inflammation and neuronal damage.

#### 5.1.1. T-Cells

The T-cell-mediated autoimmune response, crucial for initiation of MS-related CNS injury, is also relevant for progressive types of disease. The T-cell activation marker sCD27 was found to be elevated in the CSF of PPMS patients [23].

On the contrary to early stages of MS (when CD4+ T-cells are mostly implicated), CD8+ T-cells are the predominant subset found in chronic lesions, and their presence correlates with the degree of axonal damage [19,24,25,26]. Furthermore, Th1 and Th17 cells are involved in chronic inflammation underlying progression via interaction with astrocytes and microglia (promoting its proinflammatory phenotype), the upregulation of cytokines (e.g., IL-17) and the inhibition of neurotrophic factor production [27,28].

#### 5.1.2. B-Cells

Recently, it has been proposed that the interplay of T- and B-cells is a core feature of MS pathogenesis, especially in progressive types of disease [19,29]. Different mechanisms associated with B-cell activity in CNS include the production of auto-antibodies targeted against myelin, antigen presentation to Th17 and Th1 cells and driving the autoproliferation of brain-homing T cells, the secretion of inflammatory cytokines and chemokines (e.g., IL-6, il-12, IL-15, TNF-α, and INF-γ), as well as the secretion of soluble toxic factors that induce oligodendrocyte and neuronal injury [24,29,30]. It was also suggested that B-cells of MS patients are less capable of downregulating immune responses due to their deficiency in IL-10 production [29]. The depletion of B-cells using anti-CD20 monoclonal antibodies resulted in significantly diminished pro-inflammatory responses of CD4+ and CD8+ T-cells as well as myeloid cells, and provided evidence of the use of these agents as highly effective therapies both in relapsing and progressive MS.

The retention of pro-inflammatory B-cells within CNS is supposed to be mediated by activated leukocyte cell adhesion molecule (ALCAM) and their activity by B-cell maturation antigen (BCMA) and transmembrane activator and CAML interactor (TACI), two survival receptors liberated into CSF [29,31].

B-cells also contribute to the pathology of MS by producing autoantibodies against different CNS antigens presented on neurons, oligodendrocytes and astrocytes. These antibodies, reactive against myelin and other CNS antigens, can be detected as already mentioned OCB in CSF [32].

Furthermore, B-cells have been recognized as the main component of ectopic lymphoid follicles localized subpial or adjacent to the cortex, identified in brain tissue samples from subjects with progressive MS, and associated with a loss of cortical neurons and activation of microglia. Although these findings seemed more typical for a secondary progressive type of disease (and presumably for its longer duration), subpial and cortical inflammatory lesions were often demonstrated in MRI in PPMS patients [33].

#### 5.1.3. Microglia

Microglia represent a specialized population of macrophage-like cells in the CNS, acting as mediators between the peripheral and CNS immune system and creating a microenvironment for neurons via interaction with astrocytes and oligodendrocytes.

In physiological conditions, microglia provide trophic support for neurons and promote repair processes, while in the course of MS (particularly during its progressive stage), there is a transition from a homeostatic state of microglia to their altered phenotypes, dynamically changing in morphological and functional aspects in response to pathological conditions [34,35,36]. This detrimental phenotype of microglia is responsible for neuroaxonal damage via different mechanisms, linking inflammation and degeneration [36]. The presence of such activated microglia (also assigned as “microglia inflamed in multiple sclerosis—MIMS”) has been demonstrated in brain tissues from patients with progressive MS in chronic demyelinating lesions (especially smoldering/slowly expanding ones) [24,35,37], as well as within diffuse alterations in normal-appearing white and grey matter [37]. MIMS contains the two main clusters: the first one is characterized by lipoprotein reactivity and associated with lysosomal activity, the clearance of myelin debris and the regulation of the inflammatory response. The second one, with typical accumulation of iron, plays a role in antigen presentation (causing restimulation of autoreactive memory T-cells) and the propagation of inflammation (as a source of complement components, mediating antibody-dependent cytotoxicity) [37,38]. The proinflammatory effect is also associated with the production of cytokines, e.g., tumor necrosis factor-alpha (TNF-α) and interleukin 1 beta (IL-1β), found in extracellular vesicles released from activated microglia in progressive MS [39]. The chemoattraction of immunocompetent cells from the periphery and positive feedback interaction between microglia and astrocytes further enhance the inflammatory response in CNS [36].

Microglia-derived cytokines provide a link between inflammation and neurodegeneration, i.a., by facilitating glutamatergic transmission and potentiating excitotoxicity—another detrimental effect of altered microglia upon neurons (increased vulnerability due to dysregulation of ion homeostasis and metabolic intermediates) [36,40]. Moreover, excessive iron accumulation in activated microglia may promote the transition of T-cells to their pro-inflammatory and pathogenic phenotype, as well as lead to iron-mediated cell death, i.e., ferroptosis [41,42]. In addition, due to metabolic pathways associated with an increased production of ATP, altered microglia become a source of reactive oxygen and nitrogen species, contributing to oxidative stress (OS) and mitochondrial dysfunction. Iron may further enhance oxidative damage in the presence of ROS produced by the oxidative burst [41,42]. These processes, together with impaired antioxidant mechanisms, result in an increase in energy demand from neurons, with concomitant insufficient ability to supply it [36,40]. OS-mediated mitochondrial dysfunction is supposed to affect the activity of enzyme complexes and promote apoptosis [43]. It has been demonstrated in experimental models that CSF from patients with PPMS applied to healthy rat neurons induced mitochondrial dysfunction, energetic failure and axonal damage with neuronal apoptosis, which was inhibited by caspase inhibitors [41,42,43,44,45,46,47,48,49,50,51,52]. Other pathways involved in neuronal loss in progressive MS include the activation of necroptosis signaling in cortical neurons and the induction of apoptotic cascades by microglia-derived inflammatory and cytotoxic mediators (TNFα, IL-1β, IL-6 and ROS) [50].

Apart from the aforementioned mechanisms of neuronal injury, energetic deficit, lack of growth factors and cytotoxic mediators from activated microglia may specifically impair axonal growth and cause the degeneration of synapses (synaptopathy), as well as (coupled with inadequate clearance of myelin debris) contribute to the failure of remyelination [36,53].

#### 5.1.4. Astrocytes

Astrocytes provide structural and metabolic support for neurons, control the neuronal microenvironment, and participate in the immunomodulation and regulation of BBB function [54].

Similarly to microglia, in the course of progressive MS, astrocytes become activated (gathering at the edge of chronic active lesions, particularly in the cortex), which is indicated, i.a., by the upregulated expression of glial fibrillary acidic protein (GFAP). Activated astrocytes undergo a range of transcriptional and functional alterations [55], including the dysregulation of their homeostatic functions (glutamate uptake, lactate shuttling and elimination of potassium). Furthermore, astrocytes secrete cytotoxic factors, mainly associated with OS (reactive oxygen species and nitric oxide). Adverse effects of excitotoxity and OS-mediated injury comprise the impairment of neuronal metabolism and damage to the axonal cytoskeleton [36,54].

Reactive astrocytes also promote inflammation in CNS—directly (by the production of interleukins, e.g., IL-6) or chemoattracting immunocompetent cells (e.g., via CCL2). They support the survival and activity of B-cells (via B-cell-activating factor—BAFF), which reciprocally maintain the proinflammatory activation of astrocytes [36]. Moreover, microglia–astrocytes crosstalk plays an important role in the regulation of inflammatory signaling within a CNS environment [38,56].

Astrocyte alteration in progressive stages of MS may contribute to a subtle, chronic dysfunction of BBB, supposed to compromise protection of the CNS from peripheral toxic factors. In addition, the formation of a glial scar by astrocytes (isolating areas of MS-related damage) can inhibit remyelination and axonal regeneration [40,54].

### 5.2. Remyelination Failure

The loss and dysfunction of oligodendrocytes (OL) and their precursors (OPC), responsible for the production of myelin in CNS, is crucial for the balance between the processes of myelin injury and repair in the course of MS. The results of this impaired balance can be particularly observed in chronic active or “smoldering” demyelinating lesions. A range of extrinsic and intrinsic factors have an adverse impact on OPC migration, proliferation and differentiation [57].

As mentioned above, remyelination may be impeded by OL/OPC interactions with microglia and astrocytes on multiple levels (including the effect of released mediators, shaping extracellular matrix, insufficient amount of trophic support or inadequate clearance of myelin debris). Glutamate excitotoxicity and proinflammatory cytokines may directly cause the loss or dysfunction of OL, while chemokines mainly modulate the migration and proliferation of OPC [36,50,56,58]. OL and OPC, due to their poor antioxidant defense, are most susceptible to adverse effects of OS, including their selective damage [41].

Relevant impact upon the impaired proliferation and differentiation of OPC into OL is associated with aging. Aging-related alterations, displayed in animal models of demyelination, include the dysregulation of transcription factors, decreased expression of metabolic activity and the accumulation of cells with particular senescence-associated secretory phenotypes (SASPs) [59]. Neural progenitor cells (NPCs) (derived from induced pluripotent stem cell lines) acquired from PPMS patients or from white matter chronic lesions in autopsy brain tissues failed to promote OPC maturation and showed the expression of high-mobility group box-1 (HMGB1), which are considered cellular senescence hallmarks [60].

The investigation of dynamic changes in the chronic lesion microenvironment, as well as determining the endogenous components required for OL lineage cell progression, would enable a better understanding of remyelination regulation with potential therapeutic targets [57].

## 6. Monitoring and Predicting Progression in PPMS—Potential Biomarkers

One of the “hot topics” in MS research, especially with regard to progressive types of disease, is associated with putative biomarkers, useful for diagnostic, prognostic and monitoring purposes. Reliable and sensitive tools seem necessary to identify patients with higher risks of disease progression and to follow-up its course, including the assessment of the response to treatment.

### 6.1. Clinical Biomarkers

The Expanded Disability Status Scale (EDSS) [11] is the basic tool for the quantification of MS-related disability, most commonly used in routine clinical practice and as the primary outcome measure in clinical trials. An increase in the EDSS score (persistent or continuing over time) can reflect sustained disability worsening or PIRA. Although standardized, relatively simple and time-saving, EDSS has its limitations, particularly as a measure for early/silent progression. In spite of the detailed subscales evaluating different CNS functional systems, the motor function/gait ability remains the major factor affecting the total EDSS score and defining its milestones (limited walking distance and need for assistive devices). Thus, the EDSS score appears less sensitive to other disability aspects, both in patients within low and high ranges. To overcome this and to provide measures for particular aspects of disability, more refined tests are proposed, evaluating, e.g., manual dexterity, visual acuity and—particularly important—cognitive performance [61,62].

The timed 25-foot walk (T25FW) is considered one of the best-characterized measures of walking in MS, frequently used in the assessment of mobility impairment. The nine-hole peg test (NHPT) is a standard quantitative test of upper extremity function, applied for dominant and non-dominant hands, with a 20% change in the test score considered relevant for clinically meaningful worsening. Koch et al. determined the significance of progression rates in PPMS patients based on EDSS, T25FW and NHPT scales over a 3-year follow-up. Their results showed significant worsening in the EDSS and T25FW results, while only a few patients worsened in the NHPT. Although NHPT seems less appropriate as an individual primary endpoint in PPMS, it might be useful as the additional measure for the individual assessment of patients’ disability [63].

Considering the importance and frequency of cognitive failure in PPMS, adequate neuropsychological tests are indispensable in its recognition and quantification. Particular tools can be applied to evaluate performance in selected cognitive domains (e.g., California Verbal Learning Test (CVLT2) [64] for verbal memory, and Symbol Digit Modality Test (SDMT) [64,65] for attention and executive function) or Paced Auditory Serial Addition Test (PASAT), evaluating calculation ability, but also the speed and flexibility of information processing. Neuropsychological tests batteries, dedicated to MS patients, have been also developed for the more complex evaluation of cognitive performance (e.g., Brief International Cognitive Assessment for Multiple Sclerosis (BICAMS) or Brief Repeatable Battery of Neuropsychological Tests (BRB-N) [65].

Furthermore, integrated measures of physical and mental aspects of disability have been designed. The Multiple Sclerosis Functional Composite (MSFC) consists of T25FW, 9HPT and PASAT. The MSFC has been proven to be a sensitive tool for measuring the accumulation of disability throughout the disease, and is thus potentially useful in monitoring progression [8,64,65]. Similarly to the status of NEDA (“No Evidence of Disease Activity”), used as the primary outcome in clinical trials, NEP (“No Evidence of Progression”) has been proposed, defined as the absence of 24-week sustained clinical progression (measured by an increase in EDSS score; ≥20% increase in 25FWT; and ≥20% increase in 9HPT) [8,64,65].

Recently, increased attention has been paid to patient-reported outcome measures (PROMs), which provide patients’ perspective of their functioning in different spheres of life and therefore allow the evaluation of the impact of their disease. PROMs can capture particular domains, including depression, anxiety, pain, bladder control, fatigue or sleep disturbances, as well as more general issues, including overall disease burden, quality of life or social participation (e.g., PROMIS 29 questionnaire, MS Quality of Life—54-MSQOL). Due to the guidelines of EMA, these measures are being increasingly used in research and randomized clinical studies.

In multi-center three-phase trials with ocrelizumab in PPMS patients (ORATORIO, CONSONANCE), the main outcome measures included the composite evaluation of progression (NEP), SDMT and Brief Visuospatial Memory Test—Revised for the assessment of cognitive performance and 36-Item Short-Form Health Survey as a measure of health-related quality of life [66,67,68].

Regular and quantified monitoring of performance in the functional tests and PROMs seems essential for monitoring progression. Due to the increasing popularity of digital biosensors with easily administered applications (smartphones or smartwatches), frequent objective assessment in daily functioning and relevant review of the measures at follow-up visits becomes more accessible.

### 6.2. Biochemical Biomarkers

Among body fluids biomarkers of activity or progression in MS, the most relevant evidence supports the use of serum neurofilament light chain (NfL) and glial fibrillary acidic protein (GFAP).

NfL is a cytoskeletal protein that is released after axonal damage to extracellular space, penetrating to CSF and (in a much smaller but proportionate amount) to peripheral blood. Its concentration can be reliably measured both in CSF and serum [69,70,71]. Elevated NfL amount in MS patients is associated with recent inflammatory activity. However, as a sensitive marker of axonal loss, NfL can also be applied for monitoring progression. NfL concentration was found to correlate with EDSS score and its increase over time [72,73,74]. Used as the outcome measure in clinical trials with PPMS patients (INFORMS—fingolimod, ORATORIO—ocrelizumab), NfL level decreased during several months of treatment, parallel to clinical effects (slowed rate of disability accumulation) [72,73,74]. Furthermore, the predictive value of this potential marker has been shown in PPMS patients treated with high-efficacy therapies; high levels of NfL at baseline predicted the risk of confirmed disability progression or occurrence of PIRA within few years of follow-up [74].

GFAP is a monomer intermediate filament protein, mainly expressed in astrocytes; its breakdown products reflect astrocyte injury and reactive astrogliosis. GFAP seems to be an even better indicator of progression than activity, as its level not only clearly differentiates between MS patients and healthy controls, but is also significantly higher in progressive forms and inactive stages of disease [73,74]. Furthermore, in progressive MS, an elevated level of GFAP correlates with disease duration, EDSS score and its dynamics, as well as with decreased white and gray matter volumes.

Concomitant analysis of serum GFAP and NfL shows their significant relationship with each other, and increases the predictive value of these combined markers with regard to confirmed disability worsening and PIRA [70,71,72,73]. On the other hand, little specificity of both NfL and GFAP (their increased concentration occurs in a range of CNS diseases with various etiology–inflammatory, degenerative, metabolic, post-traumatic, etc.) as well as a lack of a consistent measurement methodology and normative values are limitations to their use as biomarkers in this field.

Recent studies on biomarkers of progression in MS focused on chitinase -3-like-1protein (CHI3L1). CHI3L1 is expressed, i.a., in neutrophils, macrophages, endothelial cells, astrocytes and microglia, and is involved in tissue remodeling and modulating inflammation. This secretory protein can be detected in both CSF and serum. The studies revealed elevated levels of CHI3L1 in CSF in MS patients compared to healthy controls, and in PPMS in comparison with RRMS [75]. Correlations were also found between increased levels of CHI3L1 and baseline EDSS score as well as the subsequent progression of disability, with less of a relationship with age and disease duration [75,76,77,78].

Studies on putative biomarkers associated with OS concerned, i.a., total oxidation status, level of 8-iso-prostaglandin, malondialdehyde or superoxide dismutase (SOD) as indices of pro-oxidative mechanisms. Total antioxidant status (TAS), glutathione levels and glutathione peroxidase activity have been investigated in turn as indices of antioxidant capacity [51,78]. The findings from investigations of OS indices in body fluids from MS patients suggest a disturbed balance between pro- and antioxidant mechanisms, to the detriment of the latter [43,51]. With regard to indices relevant for the progression of disease, increased level of 8-iso-prostaglandin, the product of lipid peroxidation of arachidonic acid, was demonstrated in the patients with progressive types of MS. Platelet hemostatic function, also found to be advanced in progressive MS, positively correlated with increased production of the superoxide radicals, as well as with the level of disability. The levels of advanced oxidation protein products and nitric oxide metabolites were higher in the MS subjects with a greater increase in disability over 5 years of follow-up [37,52].

Sphingolipids also seem promising candidates for the role of the markers for progression in MS. The accumulation of ceramide derivatives has been demonstrated in MS lesions and CSF, and their level correlated with clinical deterioration in progressive types of the disease. Ceramide was also suggested as a factor produced by the meningeal inflammatory infiltrates and associated with microglia activation. Sphingolipids are supposed to impair balance between signaling pathways involved in neuronal damage and neuroprotection, as well as oligodendrocyte homeostasis [3,79].

In recent years, there has been increasing interest in extracellular vesicles (EVs)—small structures released by potentially all cell types, carrying a range of molecules and acting as mediators of intercellular communication—as potential biomarkers in neurodegenerative and autoimmune diseases, including MS [80]. EVs can be revealed in all body fluids, including plasma, serum and CSF, and evaluated by means of so-called liquid biopsy [81,82]. In the microenvironment of neuroinflammation, the amount of EVs produced by immune cells increases, indicating inflammatory activity [83]. EVs released by T-cells are able to activate endothelial cells and monocytes, while EVs derived from endothelial cells in turn activate CD4+ and CD8+ T-cells [84]. In addition, EVs seem to be involved in endothelial dysfunction and BBB disruption; the amount of CNS endothelial-derived EVs has been suggested as an indicator of BBB permeability and integrity in the course of MS [85]. Furthermore, as already mentioned, EVs derived from activated microglia have a distinct proteomic profile and carry cytokines like TNF-α and IL-1, acting as mediators propagating inflammation, as well as preventing remyelination [83]. Thus, EVs released by microglia and other CNS-resident cells have been proposed as markers of these cells’ activity in vivo, which could be directly quantified in liquid biopsy from CSF by flow cytometry [86,87,88].

In the investigation of the molecular basis for the MS background, attention has been recently paid to the role of non-coding RNAs (ncRNAs), including microRNAs (miRNAs) and long non-coding RNAs (lncRNAs) [89]. miRNAs are small single-stranded molecules that regulate gene expression at the post-transcriptional level, relevant for numerous cellular processes (including apoptosis or stress response) [89,90,91]. Long (more than 200 nucleotides) non-coding RNAs (lncRNAs) take part in the regulation of transcription, post-transcription, and translation [86,92] and have a substantial impact on the development of the immune system and nervous tissue. The dysregulation of lncRNAs has been associated with processes of neuronal death in the course of neurodegenerative diseases [93]. A number of aberrantly expressed miRNAs and lncRNAs have been identified in body fluids (plasma, serum, CSF) and CNS cells from brain tissues in MS patients. Preliminary findings of these investigations suggested the possible role of ncRNA as biomarkers differentiating the progressive from the relapsing–remitting phenotype of disease or predicting the severity of its course [94,95]. Further studies using in vitro or in vivo models seem necessary to verify the relevance of these observations.

### 6.3. Radiological Biomarkers

As stated above, the number and load of new/active lesions in standard MRIs of the brain and spinal cord, which may occur especially in the early stage of PPMS, are considered appropriate markers for MS activity. However, identifying and monitoring progression requires other, more specific radiological indices. Evidence-based data support mainly the use of the following: slowly expanding lesions (SELs), paramagnetic rim lesions (PRLs), cortical lesions, and measures of global and regional CNS atrophy.

**Figure 2:**
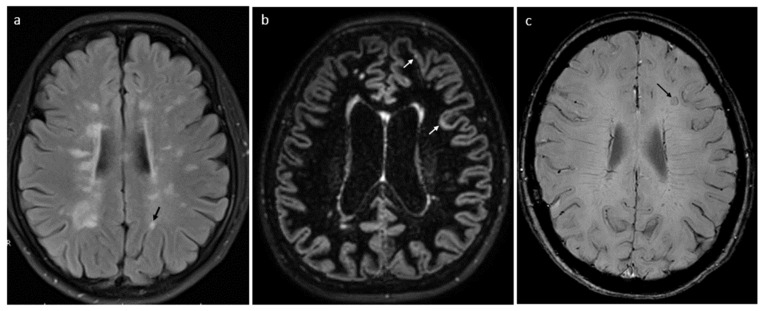
Patient with PPMS. MR images of the brain showing the following: (**a**) multiple demyelinating plaques in periventricular and juxtacortical (arrow) locations on FLAIR sequence, (**b**) cortical plaques (arrows) on DIR sequence and (**c**) a paramagnetic rim lesion (arrow) on SWI sequence; own resources.

SELs and PRLs correspond with process of multifocal, chronic CNS inflammation, a substantial compound of background for progression in MS. SELs are characterized by constant and concentric volumetric expansion in T2, with concurrent reduction in T1-weighted sequence, revealed in two to three subsequent MRI scans [22,96,97,98,99]. PRLs have a typical rim surrounding at least 75% of lesion, which reflects the layer of iron-laden microglia and macrophages, accompanying demyelination and axonal transection and can be shown in phase imaging, SWI or multi-gradient echo sequences (Figure 2).

SELs and (even more) PRLs are usually scarce in number but more frequent in progressive MS.

Their presence and amount show significant correlation with degree of disability and cognitive impairment and their deterioration over time. Furthermore, the co-localization of SELs and PRLs is strongly associated with the prediction of PIRA within further few years of follow-up [96,100].

Chronic neuroinflammation in the form of subpial infiltrates of lymphocytes, together with retrograde degeneration from axons transected within white matter lesions, contribute to cortical demyelination. Until recently, cortical lesions have been difficult to reveal by standard MRI sequences. New techniques with the use of ultra-high-magnetic field resolution (>7 T), double inversion recovery (DIR) and phase-sensitive inversion (PSIR) allow the visualization of cortical damage and ribbon-like subpial demyelination [96,101,102,103]. They appear to be more common and extensive in progressive MS and are mainly localized in the parietal lobe, followed by the frontal, occipital, temporal lobes and insula. Their total volume correlates with the level and deterioration rate of physical disability and cognitive performance [96,101,104].

**Figure 3:**
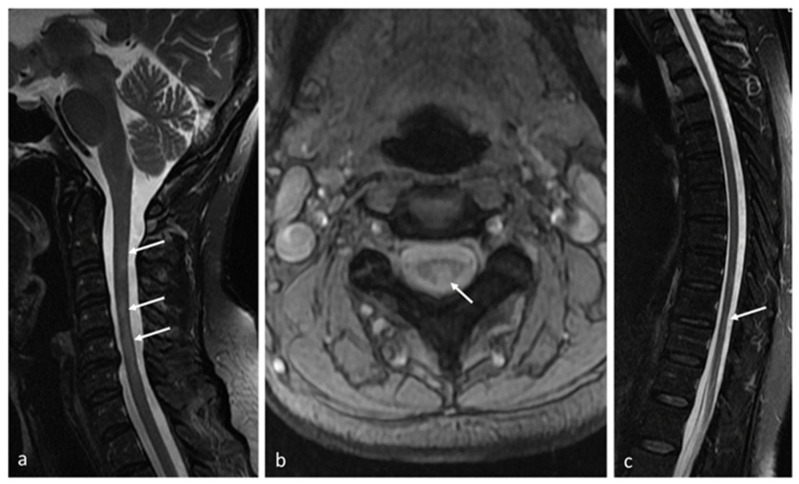
Patient with PPMS. MR images of the spine showing: (**a**) multiple demyelinating plaques within the cervical spinal cord (arrows) on a sagittal fat-saturated T2 weighted image and (**b**) in the axial plane on a T2-weighted image (arrow). Fat-saturated T2-weighted image (**c**) showing atrophy of the lower thoracic spinal cord in the course of the disease (arrow); own resources.

Cerebral atrophy is a hallmark of diffuse neurodegeneration with irreversible axonal and neuronal loss, particularly relevant in progressive MS. Increased brain volume loss in PPMS significantly correlated with disability progression, independent of the number of previous or new T2 lesions [2,51,100]. Relationships were shown between global and regional cortical atrophy and cognitive functioning, as well as fatigue and mood disorders in patients with progressive MS. Volumetric analysis of serial MRIs in PPMS revealed the highest rate of atrophy for cortical but also deep grey matter structures (including thalamus, putamen, hippocampus and cingulate gyrus). Atrophy of the thalamus has been considered as the most important measure to predict clinical worsening (including cognitive decline) within a few years’ observation [97,100,101,105]. Moreover, spinal cord volume loss is another marker of disease progression and seems to be independent of the number of spinal cord lesions (Figure 3). Spinal cord atrophy occurs at higher rates than cerebral atrophy, particularly in progressive forms of disease, and correlates with increase in disability [103] (Figure 2 and Figure 3).

Diffuse neuronal and axonal damage typical for PPMS can be depicted as “dirty-appearing white matter”—ill-defined hyperintense areas (in T2-weighted or proton density-weighted images), located mainly around the lateral ventricles. Microstructural brain tissue injury within normal-appearing white or grey matter can be even better demonstrated in diffusion MRI techniques (diffusion tensor imaging—DTI). Indices of these microstructural abnormalities—increased mean diffusivity (MD) (a measure of the degree of the restriction to diffusion) and decreased fractional anisotropy (FA)—have been shown as predictive factors for disability worsening (including cognitive performance) in progressive MS [104,106].

Another advanced neuroimaging technique, which allows the visualization of diffuse CNS damage in progressive MS, is positron emission tomography (PET). PET radioligand binding to 18kD translator protein (TSPO) corresponds with the function of activated microglia and macrophages in the brains of people with MS. Increased uptake of TSPO tracers, such as 11C-(R) -PK11195 and F- PBR111, was found in all MS phenotypes, but predominantly in progressive forms. PET turned out to also be a sensitive tool in the functional imaging of chronic demyelinative lesions (e.g., PRL) [100,101].

The integration of different radiological measures (e.g., indicating focal and diffuse MS-related CNS damage or combining structural with functional brain imaging) may provide more sensitive monitoring and predictive markers for progression in MS.

Table 1 summarizes the biomarkers of progression in MS currently in use or under investigation.

## 7. PPMS—Therapeutic Options

Ocrelizumab (OCR) is currently the only disease-modifying therapy (DMT) approved by FDA (2017) and EMA (2018) for use in the active type of PPMS (besides its application in RRMS). OCR is a humanized monoclonal antibody, targeted at CD20 antigen in B-cells. Its mode of action includes selective (excluding pro-B-cells and long-term immune memory plasmatic cells) and rapid depletion of B-cells via multiple mechanisms (apoptosis, antibody-dependent cellular cytotoxicity, antibody-dependent cell-mediated phagocytosis and complement-dependent cytotoxicity). The use of OCR in PPMS was feasible, considering the role of B-cells in chronic neuroinflammation (e.g., subpial infiltration and diffuse grey matter injury) underlying progression in MS. The results of ORATORIO, phase 3 randomized clinical trial testing OCR vs. placebo in PPMS patients, demonstrated beneficial effect of the drug on clinical and radiological measures of progression and activity of disease. During at least 24 weeks of treatment, OCR was associated with a significant reduction in the hazard for confirmed disability progression (measured with EDSS score). With regard to particular disability aspects, treatment with OCR lowered the risk of confirmed worsening in walking ability and upper limb dexterity (measured with T25FW and 9HPT scores), decreased level of physical and cognitive fatigue and improved overall quality of life [107,108,109,110,111].

Analysis of MRI parameters showed that OCR, in comparison to placebo, caused a significant decrease in total volume of T1 and T2 brain lesions, as well as lowering the rate of global and regional cerebral atrophy in the patients with PPMS, including volume of cerebellum, cerebral cortex and (most significantly) thalamus. Overall, among those treated with OCR, there was significant increase in percentage of patients who showed no evidence of progression or activity [108,109,110,111]. The safety of OCR in the PPMS cohort turned out to be similar to that of the RRMS population in the other trials, with infusion-related reactions, upper respiratory tract infections, and oral herpes infections as the most frequent adverse events, and with insignificant frequency of serious infections or malignancies.

Interesting findings from ORATORIO were associated with the use of different markers of progression and suggested their monitoring and prognostic utility. During the treatment, there was a significant difference in the decrease in serum NFl level, favoring OCR treatment to placebo. Higher baseline NFl level correlated with number and volume of SEL and with subsequent rate of reduction in thalamic volume during the treatment [108,109,110,111,112].

Long-term observations of patients from the ORATORIO cohort demonstrated that after 10 years, more than 1/3 of those continuously treated with OCR were progression-free, and more than 80% did not require a walking aid or wheelchair. Those who started the drug early maintained lower risk of confirmed disability worsening in comparison with the patients initially receiving a placebo and switching to OCR after 3 years. The safety profile of the drug in long-term data remained stable and satisfactory [112,113].

Nowadays, there are ongoing multicenter studies (eg CONFIDENCE, VERISMO or CONSONANCE), assessing the safety, adherence and persistence of OCR in newly treated MS cohorts (including PPMS ones) in a real-world setting [2,109,110,111,114].

Encouraged by these findings, another anti-CD20-antibody, rituximab, was evaluated for its efficacy and safety in PPMS in the OLYMPUS trial. Although pre-specified analyses revealed that RTX significantly slowed progression in younger patients with active plaques in MRI, the trial failed to meet its main endpoint—lowering the proportion of confirmed disability progression in the study cohort.

Unlike OCR, several other DMTs effectively used in RRMS (i.a. interferon beta, fingolimod, natalizumab, alemtuzumab) were not proved to be beneficial in PPMS. Corresponding clinical trials (e.g., INFORMS and ARPEGGIO) showed that these drugs did not significantly reduce disability progression or indices of brain atrophy in MRI [113,115].

Bruton’s tyrosine kinase inhibitors (BTKi) seem to be another promising approach for the treatment of progressive MS. BTKi exert a multidirectional effect on both innate and adaptive immune system, including activation of B-cells and activated phenotype of microglia. Furthermore, due to their small size, they are able to cross the BBB and directly modify CNS-compartmentalized inflammatory processes (with parenchymal and leptomeningeal lymphocyte infiltration), important in the progression of the disease. They are also supposed to promote myelin repair activities. Supported by the results of phase II trials, there are ongoing studies (PERSEUS for PPMS and HERCULES for SPMS) investigating the efficacy and safety of tolebrutinib in progressive types of MS [115,116,117,118,119].

Apart from targeting chronic neuroinflammation, attempts have been made to prevent progression in MS by promoting the protection of axons and myelin repair (mainly through affecting the proliferation, differentiation and maturation of oligodendrocytes).

Ibudilast is an inhibitor of cyclic nucleotide phosphodiesterases, also targeting macrophage migration inhibitory factor and Toll-like receptor 4, and penetrating through the blood–brain barrier. A phase 2 clinical trial involving patients with progressive MS demonstrated that ibudilast, in comparison with placebo, was associated with a slower rate of brain atrophy, as well as a reduced volume and magnetization transfer ratio of slowly expanding lesions (SELs). These radiological findings may indicate neuroprotective effects of ibudilast and support their further investigation [120,121,122].

High-dose biotin (HDB) activates carboxylases and enhances the production of ATP; thus, it was supposed to prevent hypoxia-driven axonal degeneration and was considered a novel therapeutic target in progressive types of MS [76]. Among disability measures in PPMS, randomized controlled trials only showed some beneficial effect of long-term intake of HDB upon walking ability, and there was strong evidence for increased laboratory test interference as a potential bias in the results [123].

Another object of investigation in this field has been the opicinumab-monoclonal antibody against LINGO-1 (inhibitor of oligodendrocyte differentiation and axonal regeneration). However, trials conducted on patients with optic neuritis or RRMS have failed to confirm the efficacy of opicinumab with regard to clinical outcomes; thus, the remyelination potential of the drug (as well as its putative impact on progressive MS) remains unclear [115,124,125].

Elezanumab is a monoclonal antibody that neutralizes repulsive guidance molecule (RGMa), an inhibitor of neurite outgrowth. In animal models of autoimmune CNS demyelination, elenazumab has been shown to decrease the area of inflammation, promote axonal regeneration and remyelination and improve functional recovery. Due to these preclinical data supporting the neuroregenerative and neuroprotective potential of the drug, the two phase 2 studies were designed to evaluate the safety and efficacy of elezanumab in patients with relapsing or progressive types of MS [126,127,128].

Oligodendrocyte precursor cells (OPC) seem a promising target for therapies stimulating remyelination. Preclinical evidence suggests a specific role for clobetasol (anti-inflammatory synthetic glucocorticoid) in the regulation of differentiation and increased viability of OPC. Studies on animal models showed that miconazole (anti-fungal, a synthetic derivative of imidazole) reduced disease activity and improved motor functionality by enhancing OPC differentiation, but did not significantly affect the remeylination. Metformine in preclinical studies appeared to have a potential impact on oxidative stress and induced the formation of new myelin [86]. There is also some evidence that muscarinic antagonists (clemastine fumarate, benztropine, and quetiapine) may exert a similar effect. Although treatment with clemastine fumarate resulted in a reduction in prolonged visual evoked potential latency (electrophysiological measure for remyelination), no radiological indices of remyelination were detected in MRI [129,130,131].

Mesenchymal stem-cell-based (MSC) therapy also has been investigated as a promising candidate for capturing multiple targets in the treatment of progressive MS. The role of MSCs is associated with the modulation of pathogenic immune responses via the production of anti-inflammatory cytokines, the inhibition of pro-inflammatory ones (by suppressing Th1 and Th17 lymphocytes) and the upregulation of regulatory processes. MSCs are also responsible for the secretion of neurotrophic growth factors, which promote the proliferation and survival of neurons, counteracting neurodegeneration and axonal loss. Moreover, MSC therapy is supposed to activate oligodendrogenesis and suppress the apoptosis of oligodendrocytes, contributing to processes of myelin repair. The results of the first placebo-controlled trial using intrathecal administration of MSCs in patients with active progressive MS demonstrated a beneficial effect of therapy upon the disease outcomes—the achievement of status without evidence of disease activity, stabilization or improvement in disability scores and decreased level of NfLs [85,102,103]. Further randomized trials are necessary to evaluate the effectiveness and a safety profile of MSC therapy in long-term observations [114,131,132].

Relevant evidence from successfully completed and ongoing clinical trials on therapeutic agents targeting progression in PPMS is summarized in Table 2.

The therapeutic approach in PPMS should also include symptomatic treatment for predominating signs of neurological deficit (e.g., relieving spasticity or bladder dysfunction), as well as physiotherapeutic interventions and psychological support (with regard to cognitive problems or mood disturbances). Due to the usually older age at PPMS onset compared to the overall MS population, aspects of CNS aging and appropriate treatment of comorbidities also have to be taken into account. Furthermore, complex therapeutic strategies, currently recommended in MS, assume the modification of lifestyle factors (e.g., balanced diet, encouraging physical and mental activity, and avoiding smoking or alcohol abuse), aimed at enhancing “neurological reserve” (the compensatory capability of CNS) and thus preventing progression [113,114].

## 8. Summary

Despite recent progress in clinical studies and the advent of the first approved therapies for PPMS (Table 3), the diagnosis and management of this type of disease still pose a challenge. In view of current concepts of “smoldering MS”, with the interplay of relapses and progression throughout the whole range of the disease course, thorough investigations of PPMS with multidimensional evaluation of progression seem necessary to elucidate the aspects not addressed so far. These investigations would provide a better insight into the processes of CNS damage underlying progression, with identification of their potential monitoring and prognostic biomarkers. Clinical implications of these findings would be associated with the reliable assessment of disease outcomes, improvement in individualized therapeutic approach and hopefully also novel therapeutic targets, relevant for the management of progression.

Table 2 summarizes chronological milestones in research on primary progressive multiple sclerosis, including treatment strategies.

## Figures and Tables

**Table 1:** Currently used or investigated biomarkers of progression in MS.

Biomarkers			Relevance	References
Clinical (repeated assessment indicates worsening in particular aspects of disability)	Functional scales	EDSS	overall degree of disability (including several functional systems)	[63]
		T25FW	mobility of gait	[63]
		NHPT	manual dexterity	[63]
	Neuropsychological tests	CVLT2	verbal memory	[64]
		SDMT	attention and executive functions	[64,65]
		PASAT	computing capacity, speed and flexibility of information processing	[65]
		BICAMS BRB-NT	cognitive performance in multiple domains	[65]
	PROMs	PROMIS 29 questionnaire, MS Quality of Life–54-MSQOL	patients’ perspective of their functioning in different spheres of life	[65]
Biochemical–body fluid (serum, blood cells, CSF)	NfL		indicates neuronal/axonal injury	[69,70,71]
	GFAP		reflects astrocyte injury and reactive astrogliosis	[73,74]
	CHI3L1		reflects chronic inflammatory activity	[75,76,77,78]
	Indices of oxidative stress	8-iso-prostaglandin	predominance of pro-oxidative over anti-oxidative mechanisms	[37,52]
		platelet hemostatic function		[37,52]
		advanced oxidation protein products and nitric oxide metabolites		[37,52]
	Sphingolipids	ceramide derivatives	indices for microglia activation, signaling pathways linking inflammation with neurodegeneration	[79]
	EVs		markers of microglia and other CNS-resident cell activity (mediators propagating inflammation and preventing remyelination)	[83]
	ncRNA		regulation of gene expression relevant for chronic inflammation and neuronal injury	[89,90,91,92,93,94,95]
Radiological (MRI)	SEL	constant and concentric volumetric expansion in T2, with concurrent reduction in T1-weighted sequence, revealed in subsequent 2–3 MRI scans	specific demyelinative lesions–sites of chronic inflammation	[96,97,98,99,100]
	PRL	typical rim surrounding at least 75% of lesion, which reflects the layer of iron-laden microglia and macro-phages, accompanying demyelination and axonal transection (phase imaging, SWI or multi-gradient echo sequences)	specific demyelinative lesions—sites of chronic inflammation	[96,97,98,99,100]
	cortical lesions	visualization of cortical damage and ribbon-like subpial demyelination (ultra-high magnetic field resolution (>7 T)	chronic neuroinflammation	[96,102,103]
	measures of global and regional CNS atrophy	loss of cerebral structure volume	diffuse neurodegeneration with axonal loss	[100,101,102,103,104,105]
	“dirty-appearing white matter”	ill-defined hyperintense areas, located mainly around the lateral ventricles	diffuse neuronal damage with chronic inflammation	[104,106]

**The abbreviations:** The Expanded Disability Status Scale (EDSS), the timed 25-foot walk (T25FW), the 9-hole peg test (NHPT), California Verbal Learning Test (CVLT2), Symbol Digit Modality Test (SDMT), Paced Auditory Serial Addition Test (PASAT), Brief International Cognitive Assessment for Multiple Sclerosis (BICAMS), Brief Repeatable Battery of Neuropsychological Tests (BRB-N), patient-reported outcome measures (PROMs), neurofilament light chain (NfL), glial fibrillary acidic protein (GFAP), chitinase-3-like-1protein (CHI3L1), slowly expanding lesions (SEL), paramagnetic rim lesions (PRL), extracellular vesicles (EVs), non-coding RNAs (ncRNAs).

**Table 2:** Evidence from completed and ongoing clinical trials on therapeutic agents targeting progression in PPMS.

Clinical Study	Description	References
2012–2016	The objective of this clinical trial was to evaluate the safety of a single intravenous infusion of autologous bone marrow-derived mesenchymal stem cells (MSCs) in multiple sclerosis (MS) with progressive disease.	ClinicalTrials.gov (accessed 3 August 2024)
- Clinical trials, Phase 1 - Completed		
2013–2017	This study reported that the treatment effect of ibudilast, in comparison with placebo, was associated with slower rate of brain atrophy in patients with PPMS.	[120] ClinicalTrials.gov (accessed 3 August 2024)
- Clinical trials (SPRINT-MS), Phase 2 - No longer looking for participants		
2014–2019	The hypothesis of this project was based on the results of animal model studies in which quetiapine fumarate was shown to have remyelinating and neuroprotective effects in models of inflammatory and non-inflammatory demyelination.	[130,131] ClinicalTrials.gov (accessed 3 August 2024)
- Clinical trials, Phase 1/Phase 2 - Completed		
2016–2016	This study confirms the efficacy of opicinumab with regard to clinical outcomes; thus, the remyelination potential of the drug (as well as its putative impact on progressive MS) remains unclear.	[115,124,125] ClinicalTrials.gov (accessed 3 August 2024)
- Clinical trials, Phase 1 - No longer looking for participants		
2017–2026	The aim of this study is to assess the safety and efficacy of Glatiramer Acetate (GA) Depot in slowing the progression of disability in patients with PPMS.	ClinicalTrials.gov (accessed 3 August 2024)
- Clinical trials, Phase 2 - No longer looking for participants - Active, not recruiting		
2020–2025	The efficacy of SAR442168 (Tolebrutinib) compared to placebo in delaying disability progression in PPMS. SAR442168, a CNS-penetrant Bruton’s tyrosine kinase (BTK) inhibitor, has the potential for a dual mechanism of action: modulation and consequent inhibition of antigen-induced B-cell activation responsible for inflammation, and modulation of macrophages and dysfunctional microglial cells linked to neuroinflammation in the brain and spinal cord.	ClinicalTrials.gov (accessed 3 August 2024)
- Clinical trials (PERSEUS), Phase 3 - Looking for participants - Recruiting		
2021–2025	An assessment of the safety of intrathecal administration of DUOC-01 cells to adults with PPMS. DUOC-01 is a population of cells isolated from donated human umbilical cord blood mononuclear cells. DUOC-01 cells are derived from CB CD14+ monocytes.	ClinicalTrials.gov (accessed 3 August 2024)
- Clinical trials, Phase 1A - Looking for participants - Recruiting		
2022–2029	Observation of patients who completed the CONSONANCE trial after a 4-year study period to assess the effect of ocrelizumab on disability such as the need to use an assistive device or a wheelchair.	ClinicalTrials.gov (accessed 3 August 2024)
- Clinical trials (CONSONANCE EXTENSION STUDY), Phase 3 - Not recruiting		
2022–2025	This study evaluates the efficacy and safety of oral masitinib, which acts as a selective tyrosine kinase inhibitor targeting immune cells (mast cells and microglia). The study involves the treatment of patients with primary progressive or secondary progressive forms of multiple sclerosis without relapsing–remitting disease.	ClinicalTrials.gov (accessed 3 August 2024)
- Clinical trials (MAXIMS), Phase 3 - Looking for participants - Recruiting		
2024–2026	Remyelination is a promising therapeutic strategy in PPMS—remote ischemic conditioning (RIC) at a dose of four cycles per day prevents gait deterioration in patients with PPMS.	ClinicalTrials.gov (accessed 3 August 2024)
- Clinical trials, phase not applicable - Looking for participants - Not yet recruiting		

**Table 3:** Chronological summary of milestones in PPMS research.

Milestone	Description	References
1996—standardized definition of PPMS	Definition of clinical course of MS, based on an international survey (USA National Multiple Sclerosis Society—NMSS). PPMS—separate subtype of disease with continuous progression from onset of clinical manifestation	[9,10,133]
2013—modified definitions of the clinical course in MS	PPMS treatment remains a separate clinical course because of the absence of exacerbations prior to clinical progression; PPMS can be additionally modified by the temporary presence of activity or progression.	[5]
2017—recent revision of McDonald diagnostic criteria	The recognition of PPMS requires the confirmation of continued clinical progression, independent of relapse activity, observed retrospectively or prospectively for at least one year, with concomitance of at least two of the following categories: ≥one lesion detected in MRI of the brain (in typical regions), ≥two lesions detected in MRI of the spinal cord, the presence of OCB in CSF.	[16]
2017–18—the approval of ocrelizumab (OCR) as the first disease-modifying therapy for PPMS	OCR-humanized monoclonal antibody targeted at CD20 antigen on B-cells. Beneficial effects of OCR on clinical and radiological measures of progression and activity in PPMS confirmed by ORATORIO clinical trial. Approved by FDA (2017) and EMA (2018) for use in the active type of PPMS.	[66,67,134]

## Supplementary Materials

The following supporting information can be downloaded at: https://www.mdpi.com/article/10.3390/ijms25168751/s1.

## Abbreviations

ALCAM	Activated leukocyte cell-adhesion molecule
APC	Antigen-presenting cells
BICAMS	Brief International Cognitive Assessment for Multiple Sclerosis
BBB	Blood–brain barrier
BCMA	B-cell maturation antigen
BRB-N	Brief Repeatable Battery of Neuropsychological Tests
CHI3L1	chitinase -3-like-1protein
CNS	Central nervous system
CSF	Cerebrospinal fluid
CVLT2	California Verbal Learning
DMT	Disease-modifying therapies
EDSS	Expanded Disability Status Scale
EVs	Extracellular vesicles
GFAP	Glial fibrillary acidic protein
lncRNAs	Long non-coding RNAs
miRNAs	microRNAs
MS	Multiple sclerosis
MSFC	Multiple Sclerosis Functional Composite
NEDA	“No Evidence of Disease Activity”
NEP	“No Evidence of Progression”
NHPT	The nine-hole peg test
ncRNAs	Non-coding RNAs
OCB	Oligoclonal IgG bands
OCGB	Oligoclonal immunoglobulin G bands
OL	Oligodendrocytes
OS	Oxidative stress
PASAT	Paced Auditory Serial Addition Test
PIRA	Progression independent of relapses
PPMS	Primary progressive multiple sclerosis
PRL	Paramagnetic rim lesions
PROMs	Patient-reported outcome measures
RAW	Relapse-associated worsening
RNS	Reactive nitrogen species
ROS	Reactive oxygen species
RPMS	Relapsing–progressive multiple sclerosis
RRMS	Relapsing–remitting multiple sclerosis
SDMT	Symbol Digit Modality Test
SEL	Slowly expanding lesions
SPMS	Secondary progressive multiple sclerosis
TACI	Transmembrane activator and CAML interactor
TAS	Total Antioxidant Status
