# Supplementary material for: Primary Progressive Multiple Sclerosis—A Key to Understanding and Managing Disease Progression
Source: Int J Mol Sci. 2024 Aug 11;25(16):8751. doi: 10.3390/ijms25168751 (PMC11354232; doi:10.3390/ijms25168751)
Supplement: Supplementary file 1 [file ijms-25-08751-s001.zip › ijms-3138594-supplementary.pdf]

2. **Method – literature search** (see: Supplemental material)

A systematic literature search was conducted of the databases Medline, Cochrane Central Register of Controlled Trials, PubMed, Web of Science and Google Scholar. The search string used was “primary pro-gressive multiple sclerosis” /“PPMS” /”progressive multiple sclerosis” and “progression independent from relapses”/ “PIRA” and “progression” /“neuroinflammation”/neurodegeneration” and “ andom-izsion”/”biomarkers”, with the hyphenation of the keywords adjusted according to the database specifica-tions. Reviews, original studies and andomized studies were selected for analysis. Abstracts and full-text manuscripts in languages other than English were excluded. References from selected articles were then screened for further records and evaluated for their relevance for the topic of the study (Fig.1). The authors independently assessed the selected articles to evaluate their eligibility, and disagreements were solved by discussion.

Figure The publication search strategy.

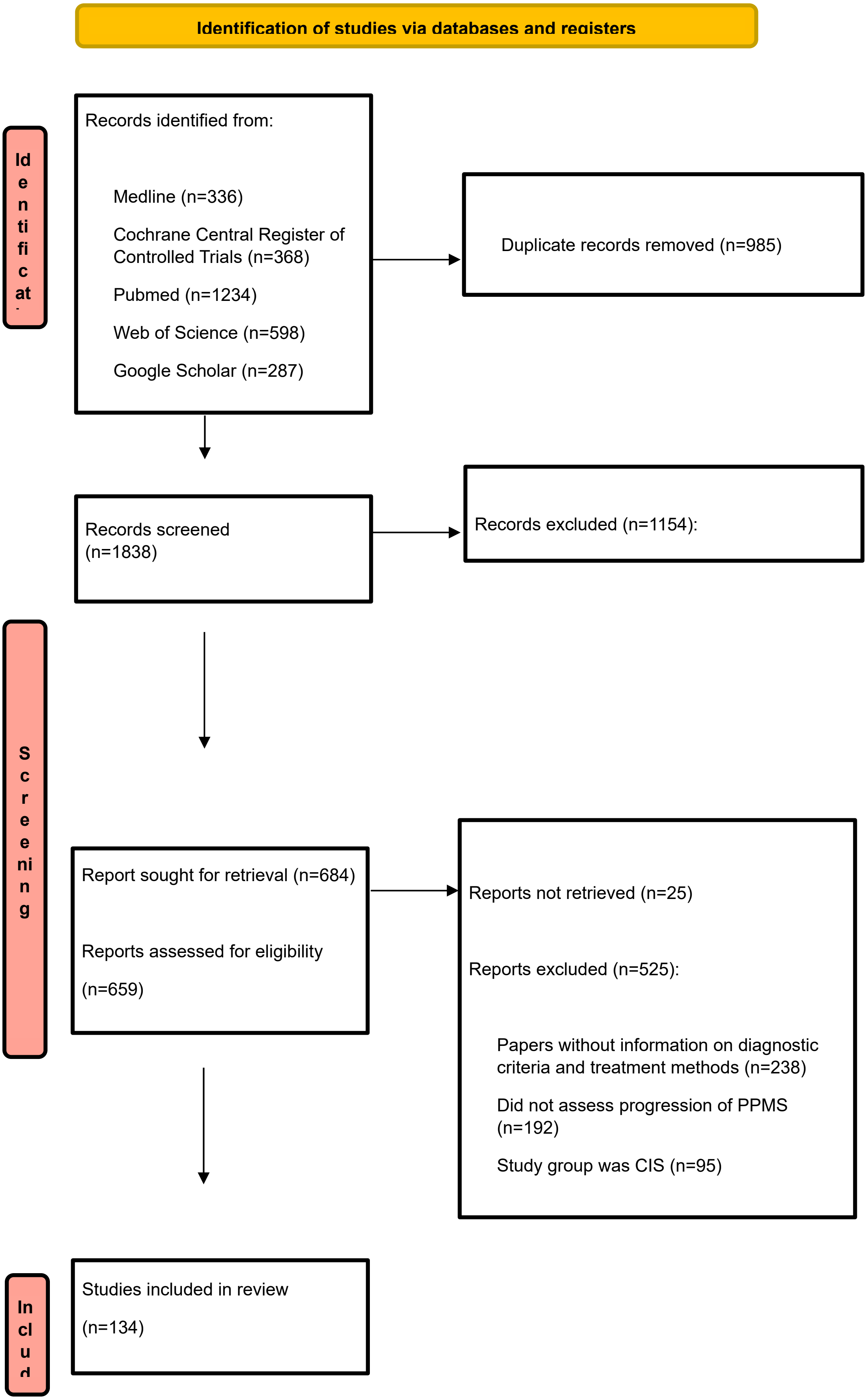

Identification of studies via databases and registers
Identificat
Records identified from:
Medline (n=336)
Cochrane Central Register of Controlled Trials (n=368)
Pubmed (n=1234)
Web of Science (n=598)
Google Scholar (n=287)
Duplicate records removed (n=985)
Records screened (n=1838)
Records excluded (n=1154):
Screening
Report sought for retrieval (n=684)
Reports assessed for eligibility
(n=659)
Reports not retrieved (n=25)
Reports excluded (n=525):
Papers without information on diagnostic criteria and treatment methods (n=238)
Did not assess progression of PPMS (n=192)
Study group was CIS (n=95)
Includ
Studies included in review
(n=134)
